# Case report: High grade serous fallopian tube carcinoma with rare *NRG1* gene fusion presenting as widespread peritoneal carcinomatosis

**DOI:** 10.3389/fonc.2024.1472725

**Published:** 2024-11-06

**Authors:** Anthony Crymes, Mark G. Evans, Tolulope Adeyelu, Jack Reid, Ifegwu O. Ibe, Matthew J. Oberley, Jill H. Tseng

**Affiliations:** ^1^ Department of Medicine, Keck School of Medicine of the University of Southern California, Los Angeles, CA, United States; ^2^ Department of Pathology, Caris Life Sciences, Phoenix, AZ, United States; ^3^ Department of Clinical and Translational Research, Caris Life Sciences, Phoenix, AZ, United States; ^4^ Department of Pathology, City of Hope National Cancer Center, Duarte, CA, United States; ^5^ Department of Pathology, Affiliated Pathologists Medical Group, Orange, CA, United States; ^6^ Department of Obstetrics and Gynecology, University of California Irvine School of Medicine, Irvine, CA, United States

**Keywords:** NRG1 gene fusion, ovarian cancer, fallopian tube cancer, primary peritoneal cancer, serous carcinoma

## Abstract

The discovery of gene fusions involving Neuregulin-1 (*NRG1*) within solid tumors has important therapeutic implications, as they are being actively explored as targets for emerging ERBB/ERBB2/ERBB3-directed anti-cancer agents. *NRG1* fusions are very uncommon across all tumor types and are infrequently documented in the medical literature. We report a female patient presenting with widespread peritoneal carcinomatosis diagnosed as high grade serous fallopian tube carcinoma, which harbored a previously undescribed *MYH10*::*NRG1* fusion. Moreover, we queried the whole transcriptome sequencing results of neoplasms analyzed by a commercial laboratory (Caris Life Sciences) to determine the overall incidence of *NRG1* fusions in carcinomas of the ovary, fallopian tube, and peritoneum (0.18%). Twenty-five additional tumors were found to demonstrate *NRG1* fusions, including 20 new genes partners that had not been previously identified in gynecologic carcinomas. Overall, *NRG1* fusion events are rare in ovarian, fallopian tube, and primary peritoneal carcinomas, but they may carry diagnostic significance in the context of borderline/low grade serous tumors, which demonstrated exclusively *CLU::NRG1* fusions, and could have important predictive implications for response to ERBB/ERBB2/ERBB3-directed therapies.

## Introduction

Neuregulin-1 gene (*NRG1*) exists as six distinct isoforms that modulate the Erb-B2 receptor tyrosine kinase (ERBB) receptor pathways to influence ERBB2/ERBB3 protein signaling via an epidermal growth factor (EGF) family protein (1). Fusions including *NRG1*, in which it typically occurs as the 3’ partner, disrupt the normal growth and differentiation processes in epithelial and other cell types, and instead constitutively activate the phosphatidylinositol 3-kinase (PI3K) and mitogen-activated protein kinase (MAPK) signaling pathways through an intact EGF domain (2). To date, several 5’ fusion genes partners have been identified, with the most frequent being *CD74* and *SLC3A2* (3). However, with new diagnostic methodologies, the list of gene partners continues to expand. *NRG1* fusions have been detected across solid tumor types with an incidence of 0.2%, with the greatest occurrence rate being observed in cholangiocarcinoma, pancreatic ductal adenocarcinoma, and renal cell carcinoma (0.5% for each) (3).

In one study of 21,858 tumor specimens (3), 0.4% of ovarian, fallopian tube, and primary peritoneal carcinomas were found to harbor *NRG1* fusions. Within these tumors, the gene was fused to *SETD4*, *TSHZ2*, and *ZMYM2*. Recent research has also reported *RAB3IL1* and *CLU* as partners (4). While rare, appropriate identification of the *NRG1* fusion is essential, as it likely represents the oncogenic driver and occurs mutually exclusive with other targetable genetic alterations (5). Because differences in testing methodology can influence reliable detection of clinically actionable fusions involving genes like *NRG1* and *NTRK1/2/3* (6), the American Society of Clinical Oncology has advocated for the use of RNA sequencing (7). Ultimately, the presence of an *NRG1* fusion may be important, in that it could predict response treatment. Our study highlights clinical course of single patient who, to-date, has experienced a good response to traditional platinum-based chemotherapy utilized during the management of gynecologic malignancies. Moreover, our work expands the spectrum of reported fusions involving *NRG1* and novel partner genes, ultimately considering the utility of ERBB/ERBB2/ERBB3-directed anti-cancer agents, such as afitinib, tarloxotinib, seribantumab, zenocutuzumab, and others, that are under investigation for the treatment of *NRG1* fusion-positive tumors (8–10).

## Methods

Paraffin-embedded tumor samples were analyzed by DNA (592-gene or whole exome) and RNA (whole transcriptome) sequencing at Caris Life Sciences (Phoenix, AZ), utilizing a customized Agilent SureSelect Human All Exon V7 bait panel (Santa Clara, CA) and Illumina NovaSeq technology (San Diego, CA). *NRG1* fusions were identified within the samples if ≥3 junction reads were detected by RNA sequencing. A board-certified pathologist (M.G.E.) evaluated those specimens with *NRG1* fusions to render a pathologic diagnosis. In order to evaluate differential gene expression present within the *NRG1* fusion-positive cases, Gene Set Enrichment Analysis (GSEA) was carried out using whole transcriptome data and the Hallmark gene set collection from the Human Molecular Signatures Database (11, 12). Transcriptomic signatures of the *NRG1* fusion-positive tumors were compared to those of previously tested low grade (728 samples) and high grade (7,818 samples) serous carcinomas of the ovary, fallopian tube, or peritoneum lacking an *NRG1* fusion.

## Results

Of the 14,395 samples obtained from ovarian, fallopian tube, or primary peritoneal carcinomas that were tested by whole transcriptome sequencing, 26 were shown to harbor *NRG1* fusions (incidence of 0.18%) between 2019 and 2023. A diversity of gene partners were identified and most were novel: *CLU* (n=4), *SARAF* (n=2), *SCAF4* (n=1), *MUC16* (n=1), *WFDC2* (n=1), *NOTCH2* (n=1), *APP* (n=1), *SPIDR* (n=1), *HGSNAT* (n=1), *SPINT2* (n=1), *RBPMS* (n=1), *JAG1* (n=1), *NRP2* (n=1), *CHMP4C* (n=1), *CXADR* (n=1), *TMEM65* (n=1), *INSR* (n=1), *ADAM9* (n=1), *LDLR* (n=1)*, TNFRSF12A* (n=1), *SPON1* (n=1), and *MYH10* (n=1). *NRG1* breakpoints were observed in either exon 2 (46%, n=12) or exon 6 (54%, n=14); exon 2 splicing preserves the gene’s immunoglobulin and EGF domains, but only the EGF domain remains intact with exon 6 splicing. All fusion-positive samples were diagnosed as borderline/low grade or high grade serous carcinoma, but one case harboring a *RBPMS::NRG1* fusion was classified as clear cell carcinoma. Of note, the *CLU::NRG1* fusion was only observed in borderline/low grade cases.

One high grade tumor was classified as tumor mutational burden (TMB)-high with >10 mutations/megabase (Muts/Mb). Additionally, three cases were notable for homologous recombination deficiency (HRD) as indicated by pathogenic variants in *BRCA1* and/or *BRCA2*, or a high genomic scar score that combines genomic loss of heterozygous (gLOH) with large-scale state transitions (LST). Those tumors with HRD were all diagnosed as high grade serous carcinoma, specifically harboring fusions involving *NRG1* and the *JAG1*, *SPON1*, and *TNFRSF12A* partner genes.

**Figure 1:**
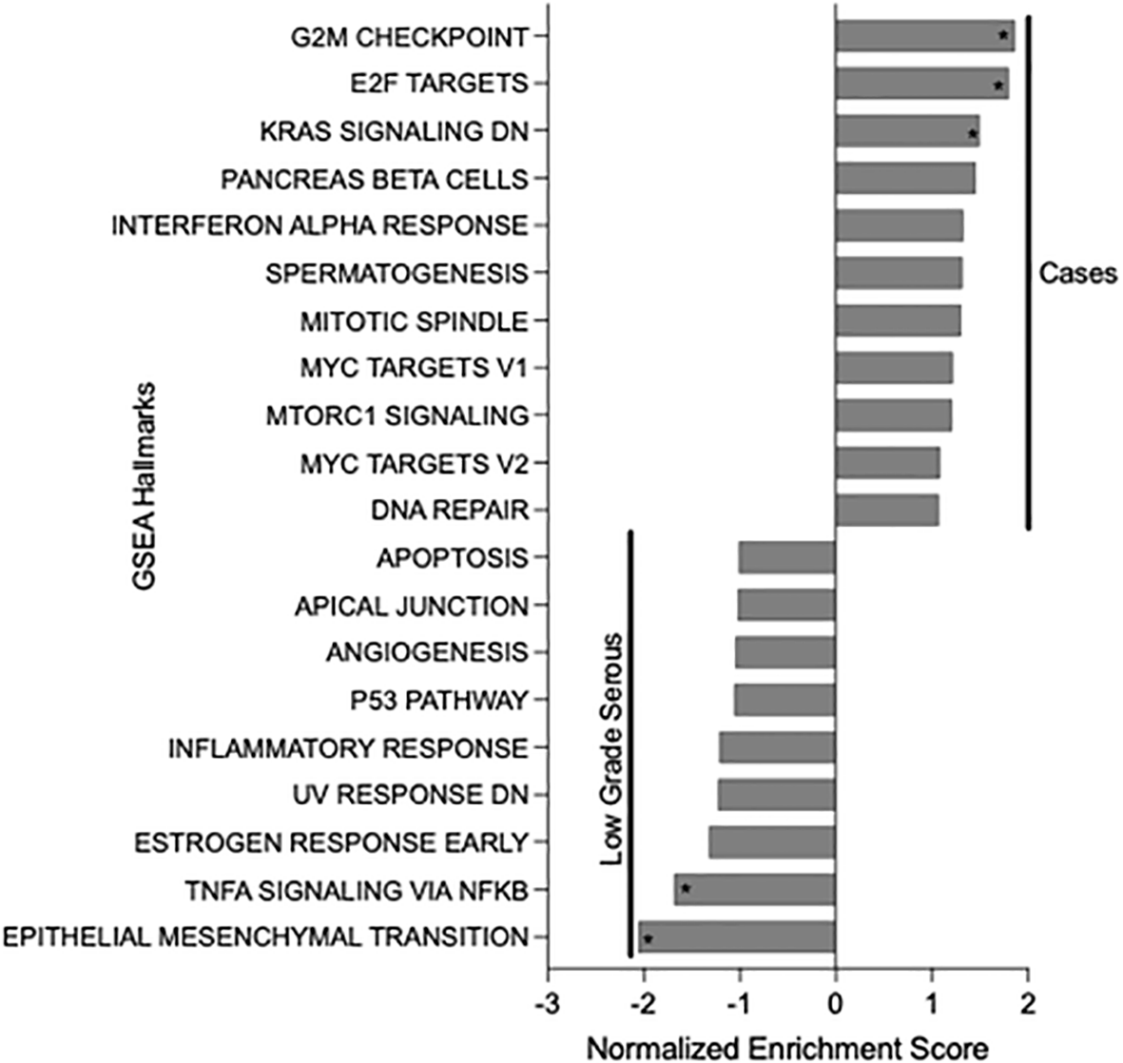
Gene Set Enrichment Analysis (GSEA) results of *NRG1* fusion-positive tumor cases compared to low grade serous carcinomas of the female genital tract. Asterisks indicate statistically significance differences (p-value <0.05).

Pathogenic genetic changes in addition to the *NRG1* fusions and HRD-associated mutations that were detected within the *NRG1* fusion-positive tumors are summarized in **Supplementary Table 1**. GSEA pathway analysis of those 26 neoplasms demonstrated that the G2M checkpoint pathway, E2F transcription factors, and *KRAS* downstream signaling were upregulated compared to fusion-negative, low grade serous carcinomas of the ovary, fallopian tube, or peritoneum (**Figure 1**). Moreover, compared to low grade serous carcinoma, the cancers harboring *NRG1* fusions demonstrated statistically lower expression of TNFA signaling genes and those associated with the epithelial-mesenchymal transition. There were no significant pathways enriched when comparing *NRG1* fusion-positive tumors with fusion-negative high grade serous carcinomas.

## Case report

**Figure 2:**
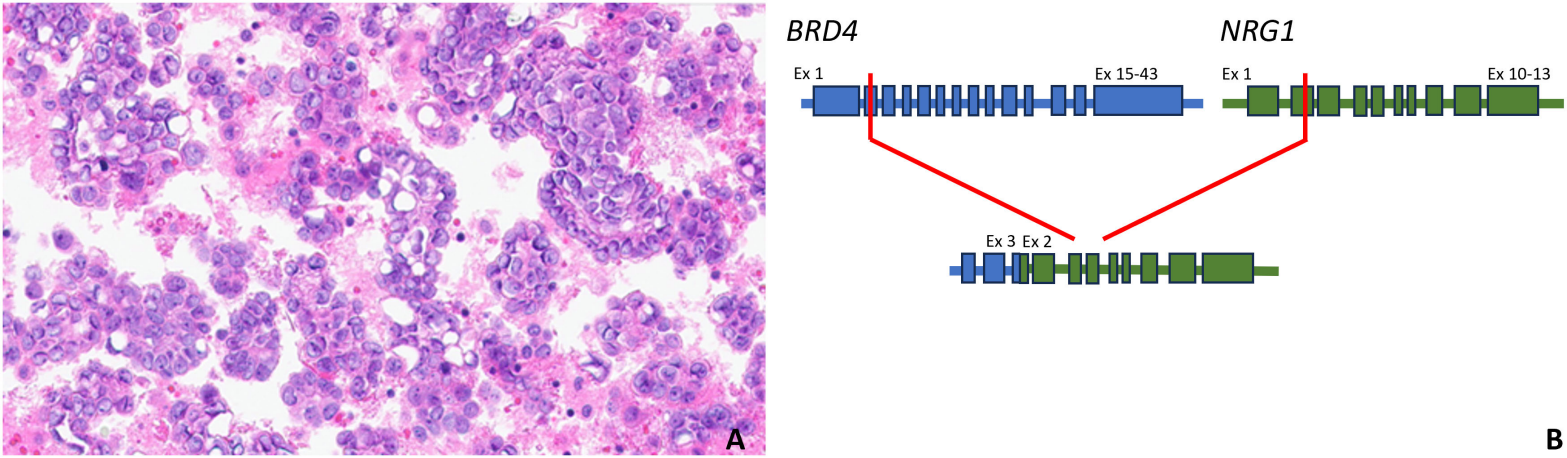
Hematoxylin and eosin-stained high grade serous carcinoma metastatic to pleural fluid harboring a *MYH10* exon 3::*NRG1* exon 2 fusion (400x magnification, **(A)**; a pictorial representation of the *MYH10::NRG1* gene fusion, which includes the intact immunoglobulin and EGF domains of *NRG1*. Ex, exon **(B)**.

**Figure 3:**
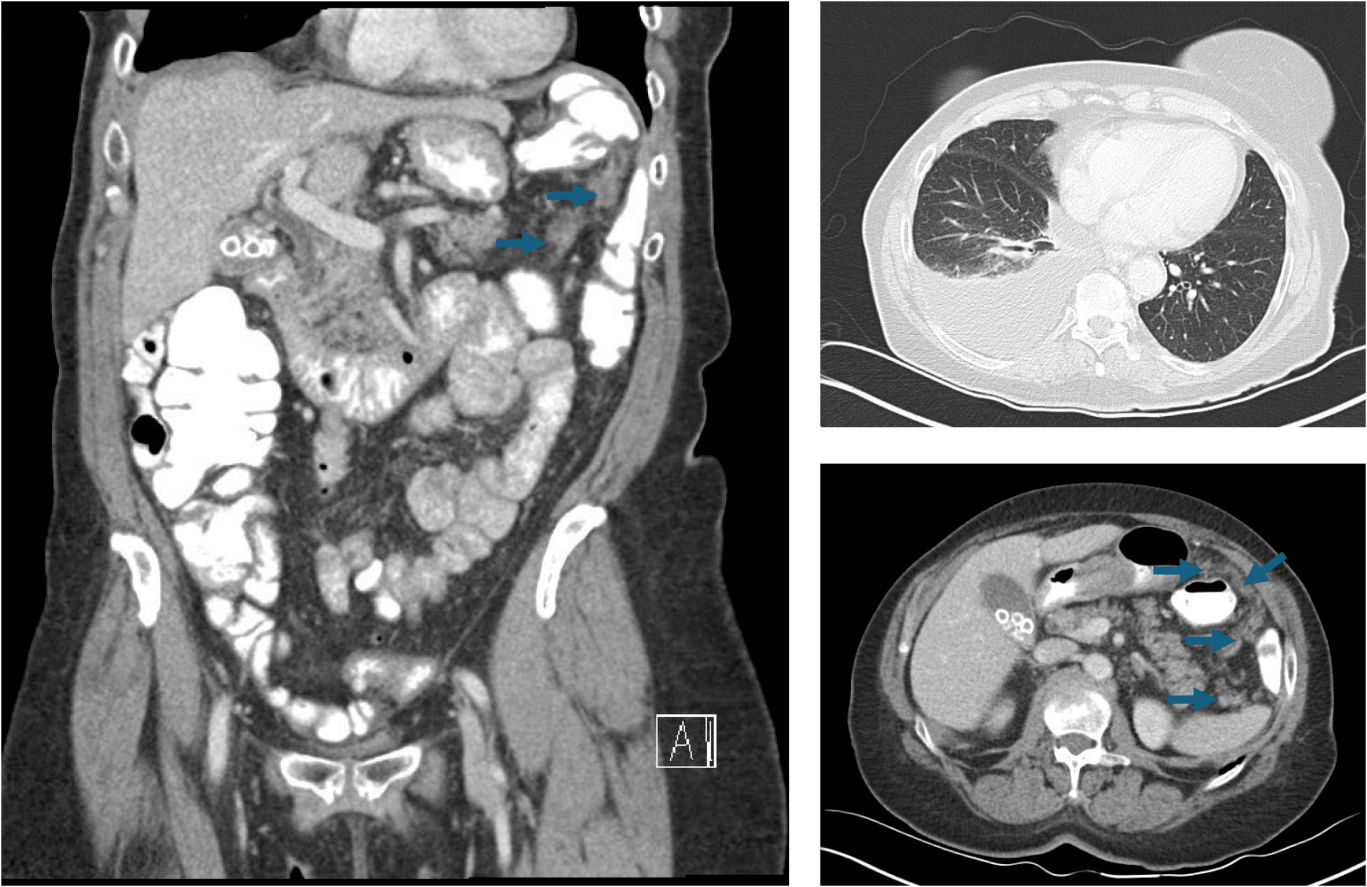
CT chest, abdomen, and pelvis imaging that reveals a right-sided pleural effusion and widespread peritoneal carcinomatosis (arrows).

A 75-year-old G1P1 woman presented to the emergency department with a several-week history of fatigue and shortness of breath. In the prior several days, she had also developed a cough and abdominal bloating that were interfering with her daily life and had become alarming. Her past medical history included essential hypertension, and she endorsed an approximately 21-year smoking history. Upon evaluation, that patient was found to have decreased right-sided breath sounds and mild abdominal distention. Her laboratory studies were unremarkable with the exception of a CA-125 level of 357 units/milliliter. A chest radiograph demonstrated right lung opacities concerning for pneumonia and a right-sided pleural effusion. Thoracentesis was performed, and the patient was surprised to learn that microscopic examination of the pleural fluid revealed scattered nests of atypical cells arranged in papillary structures, demonstrating moderate amounts of eosinophilic cytoplasm and nuclei with prominent nucleoli, pleomorphism, and mitoses (**Figure 2A**). The cells were positive for PAX8, WT1, and ER by immunohistochemistry, consistent with high grade serous fallopian tube carcinoma. Whole transcriptome sequencing identified a *MYH10* exon 3::*NRG1* exon 2 fusion (**Figure 2B**), and exomic analysis revealed *TP53* (p.K132R) and *RET* (p.C618Y) pathogenic single nucleotide variants, as well as a TMB of 3 Muts/Mb. Subsequent CT imaging demonstrated a large right pleural effusion and peritoneal carcinomatosis (**Figure 3**).

**Figure 4:**
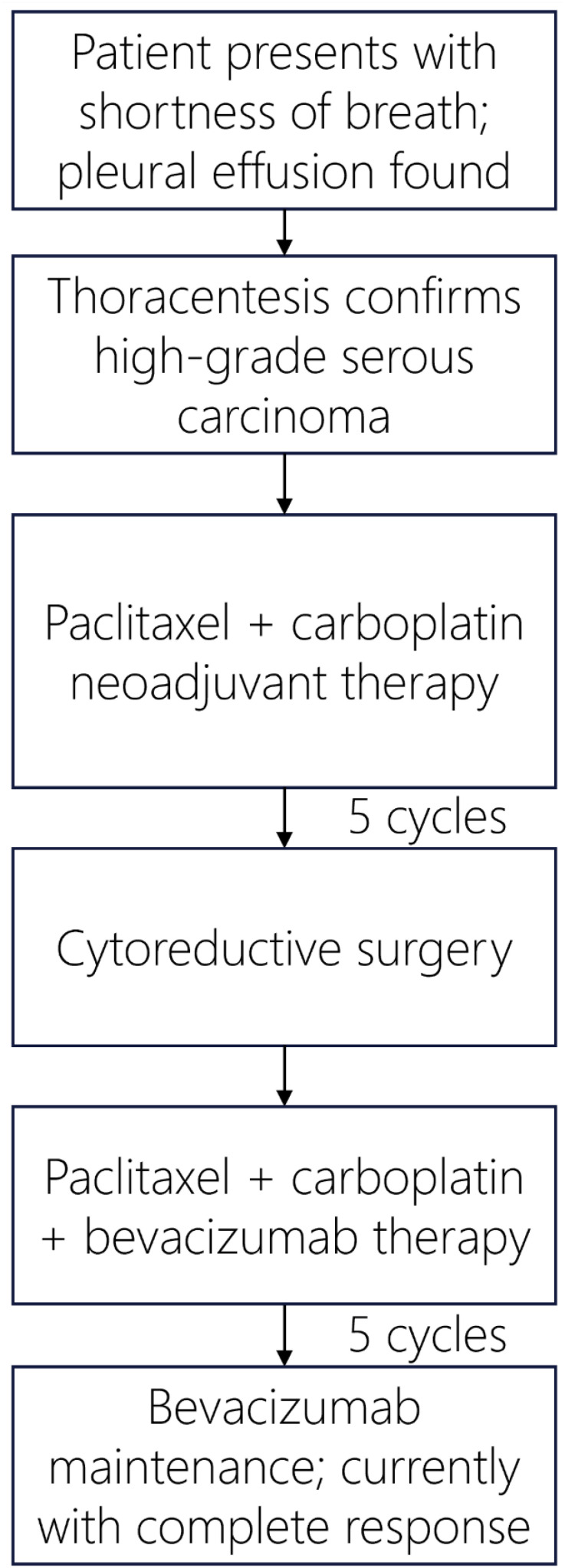
Patient’s treatment timeline for management of Stage IV high grade serous fallopian tube carcinoma.

The patient received paclitaxel and carboplatin neoadjuvant chemotherapy and developed severe abdominal pain, which indicated a large bowel obstruction for which she underwent a diverting colostomy. She received a total of 5 cycles of neoadjuvant chemotherapy prior to cytoreductive surgery that included a total abdominal hysterectomy with bilateral salpingo-oopherectomy, splenectomy, partial pancreatectomy, partial colectomy, omentectomy, cholecystectomy, appendectomy, tumor debulking and colostomy reversal. Residual high grade serous carcinoma was identified in numerous peritoneal implants, as well as within the bilateral ovaries and fallopian tubes, endometrium, cervix, parametria, pancreas, spleen, peri-appendiceal soft tissue, small intestinal mesentery, and omentum. The patient received 5 cycles of adjuvant paclitaxel and carboplatin with bevacizumab and remains on bevacizumab maintenance. Per the patient, the most challenging aspect of post-surgical treatment was persistent fatigue, which has improved significantly while receiving only bevacizumab. At last follow-up, 9 months following her final cycle of platinum-based chemotherapy, she was deemed disease-free by PET imaging. A summary of the patient’s clinical course is provided in **Figure 4**.

## Discussion

This patient’s tumor represents a high grade serous carcinoma harboring a previously unreported *NRG1* fusion; the gene is spliced with *MYH10* with an exon 2 breakpoint. Overall, our analysis demonstrates that such *NRG1*-rearranged gynecologic neoplasms are rare, with only 26 total examples identified within the total gynecologic tract cancer specimens that underwent whole transcriptome sequencing—an incidence of 0.18% among carcinomas of the ovary, fallopian tube, and peritoneum. Although uncommon, the list of genes fused with *NRG1* is extensive, with our work identifying 20 rare partners. In some instances, the specific fusion could be diagnostic. In particular, *CLU::NRG1* was only detected in cases demonstrating low grade/borderline histology. Beyond the novelty of these fusions, their gene products may have clinical significance with the advent of anti-ERBB/ERBB2/ERBB3 therapies. The efficacy of these drugs requires interaction with the immunoglobulin and EGF domains of *NRG1* (13), which have been universally retained within the fusion proteins created upon rearrangement with the various partner genes.

Moreover, in keeping with prior studies (5), those neoplasms with *NRG1* fusions described here demonstrated low TMB. Genetic changes responsible for HRD were indeed detected, although in a minority of the NRG fusion-positive samples (11.5%). While this proportion of cases is below the up to 50% prevalence of HRD reported in high grade serous carcinoma (14), these few cases could have been eligible for poly (ADP-ribose) polymerase (PARP) inhibition, which has demonstrated efficacy in treating gynecological tumors (15). Ultimately, the lack of HRD in our patient’s tumor rendered her ineligible for PARP inhibition. Whether the detected novel *NRG1* fusion and co-occurring genetic alterations (involving *RET* and *TP53*) could influence her treatment options and prognosis remains unclear.

This reality stems from the limitations of our study. By focusing on a single case, the generalizability of our patient’s experience comes into question. Moreover, the lack of extensive follow-up data does not exclude a future change in her health status. Finally, utilization of testing from a single laboratory could introduce bias in detecting and reporting the *NRG1* gene changes. Fusion confirmation could ultimately be achieved by alternative methodologies, including immunohistochemistry and fluorescence *in situ* hybridization directed against *NRG1*, although these techniques may not be specific to certain novel breakpoints and have limited commercial availability.

A comprehensive literature review summarizing diagnostic, therapeutic, and preventative strategies for treating tubal carcinomas by Colombi et al. advocates for surgical staging and debulking surgery when the patient’s physical condition allows, which would then be followed by systemic chemotherapy (16). And as would be indicated for managing our patient’s Stage IV tumor, the National Comprehensive Cancer Network (NCCN) guidelines recommend “platinum-based” regimen after cytoreductive surgical intervention (17). Similar to her personal experience, some research indicates that *NRG1* fusion-positive tumors respond to such treatment. In a study of 19 individuals diagnosed with lung carcinoma, stable disease was noted in eight whom had received chemotherapy (47%). A partial response was observed in two (12%), and disease progression occurred in eight (41%) (18). No subjects demonstrated a response to anti-PD-L1 monotherapy or combination chemoimmunotherapy.

More encouraging are the recently developed anti-ERBB/ERBB2/ERBB3 targeted therapies for the treatment of carcinomas harboring *NRG1* fusions, as would be considered for our patient if her tumor were to eventually recur. A multi-center alliance study compiling data from the management of *NRG1* fusion-positive lung cancers documented a 25% overall response rate to afatinib, a pan-ERBB small molecular tyrosine kinase inhibitor (18). While progression-free survival (PFS) was only 2.8 months, the authors advocated for afitinib in the treatment of cancers with *NRG1* fusions. A similar pan-ERBB inhibition approach is currently being explored with tarloxotinib for the management of *NRG1* fusion-positive tumors (RAIN-701 trial, NCT03805841), the results of which are expected to be reported soon (19).

ERBB3-selective inhibitors, such as seribantumab, are also currently undergoing investigation. During a Phase II clinical trial (CRESTONE trial, NCT04383210), this anti-ERBB3 IgG_2_ monoclonal antibody produced an ORR of 33% across various tumor types harboring *NRG1* fusions (20). A 92% percent disease control rate included two patients who demonstrated a complete response, thereby lending support for ERBB3-specific inhibition as a viable treatment strategy.

Recently, the United States Food and Drug Administration fast-tracked clinicals trials for a ERBB2/ERBB3 bispecific monoclonal antibody, zenocutuzumab. In a Phase I/II clinical trial (NCT02912949), this drug demonstrated excellent efficacy in treating *NRG1* fusion-positive tumors, especially those arising from the pancreas (21). A partial response was documented in 42% the pancreatic tumors, with disease progression observed in only a single case. Overall, zenocutuzumab has demonstrated a preliminary ORR of 34% across solid tumor types (22). As a result, the simultaneous targeting of ERBB2 and ERBB3 could exist as a novel approach to treating *NRG1* fusion-positive tumors. In the future, additional novel therapies are likely to be discovered, and proper assessment for changes in *NRG1* will become necessary for treatment selection.

## Conclusion

Our report of novel *NRG1* gene rearrangements detected in serous carcinomas of the ovary, fallopian tube, and peritoneum indicates a small, but important prevalence of these fusions occurring within the female genital tract. While the knowledge gained from the experience of a single patient may be limited, it describes a successful treatment strategy including neoadjuvant/adjuvant platinum-based chemotherapy, cytoreductive surgery, and bevacizumab. Moreover, the patient’s novel *NRG1* fusion highlights the potential role of anti-ERBB/ERBB2/ERBB3 therapies in managing similar tumors and illustrates the importance of diagnostic gene fusion testing.

## Data Availability

The original contributions presented in the study are included in the article/**Supplementary Material**. Further inquiries can be directed to the corresponding author.
