# Supplementary figures and images for: Case report: High grade serous fallopian tube carcinoma with rare NRG1 gene fusion presenting as widespread peritoneal carcinomatosis
Source: Front Oncol. 2024 Nov 6;14:1472725. doi: 10.3389/fonc.2024.1472725 (PMC11580015; doi:10.3389/fonc.2024.1472725)

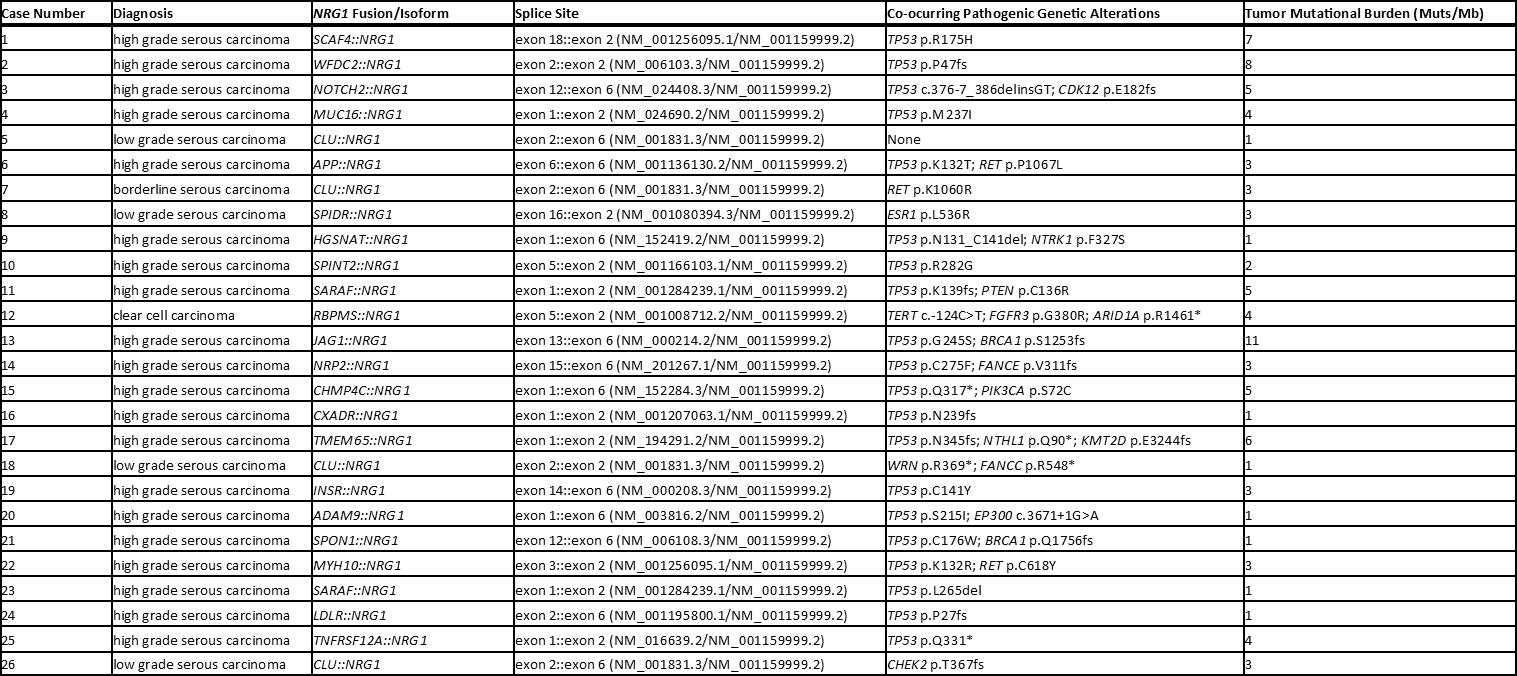

Supplement: Supplementary file 1 [file Image1.tif]
